# Case Report: Locally advanced primary esophageal melanoma with early brain metastasis

**DOI:** 10.3389/fonc.2026.1880200

**Published:** 2026-07-28

**Authors:** Huixin Yu, Shuo Sun, Xianfeng Lu, Xunjie Kuang, Yi Duan, Mingfang Xu, Xiao Yang, Mengxia Li

**Affiliations:** Department of Cancer Center, Daping Hospital, Army Medical University, Chongqing, China

**Keywords:** adjuvant therapy, case report, esophagectomy, immunotherapy, primary malignant melanoma of the esophagus (PMME)

## Abstract

**Introduction:**

Primary malignant melanoma of the esophagus (PMME) is a rare malignancy of the esophagus. The main treatment is esophagectomy, but the recurrence and metastasis rate are very high, and the prognosis is very poor. Here, we report a case of locally advanced PMME that rapidly developed intracranial metastases after surgery and adjuvant therapy.

**Case presentation:**

The patient was a 47-year-old male with dysphagia. Endoscopy revealed multiple cauliflower-like lesions in the middle esophagus, confirmed as malignant melanoma via biopsy with positive immunohistochemical staining for SOX-10 and Melan-A, with regional lymph node metastases. The patient was diagnosed with PMME and was treated with radical esophagectomy. The adjuvant therapy used tislelizumab combined with lenvatinib. Five months after surgery, follow-up magnetic resonance imaging (MRI) indicated multiple intracranial metastases. The patient underwent a tumor resection of the left cerebellar lesion, and stereotactic radiosurgery for other lesions, and received oral temozolomide. One month after radiotherapy, his condition deteriorated rapidly, showing systemic progression, and he eventually passed away ten months after diagnosis.

**Conclusion:**

Despite surgical resection and adjuvant therapy, this case of PMME rapidly progressed with multiple intracranial metastases, highlighting the highly aggressive nature and poor prognosis of this malignancy.

## Introduction

Primary malignant melanoma of the esophagus (PMME) is a rare malignant tumor

that presents without significant symptoms in the early stages (1–3), progresses rapidly and has a poor prognosis, so most patients are diagnosed at an advanced stage (4, 5). Currently, there is no standardized treatment for PMME. Surgical resection is the main treatment for early-stage PMME, but the recurrence rate after surgery remains high (5–7). In this case report, we present a patient with locally advanced PMME who rapidly developed intracranial metastases following radical surgery and adjuvant therapy.

## Case presentation

**Figure 1:**
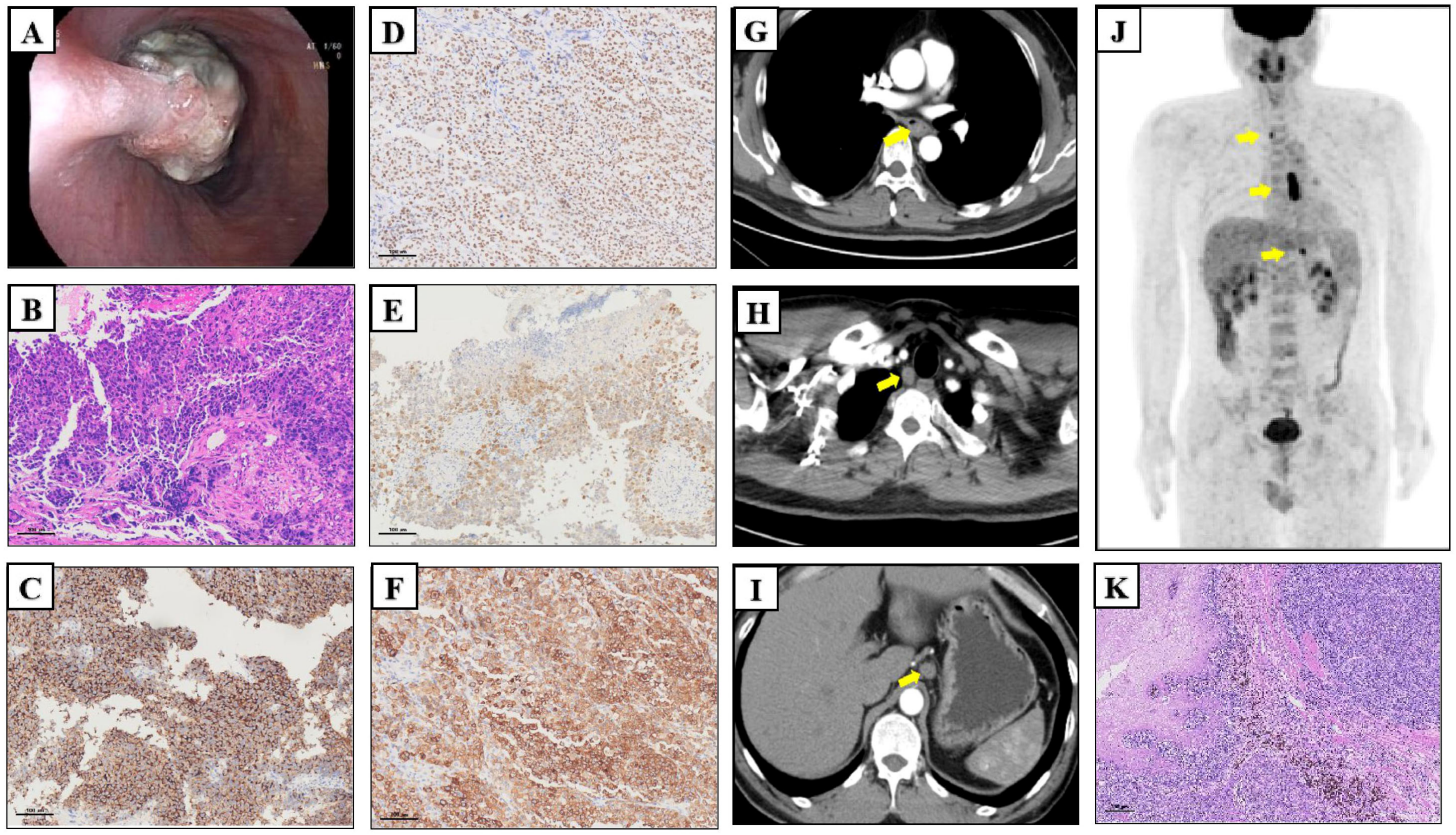
Preoperative findings. **(A)** Esophagogastroscopy showed multiple cauliflower-like lesions with pigmentation in the middle esophagus. Hematoxylin and eosin staining **(B)**, Immunohistochemistry staining of HMB-45 **(C)**, S-100 **(D)**, Sox-10 **(E)**, and Melan-A **(F)** in the biopsy samples with magnification × 40. **(G–I)** Contrast-enhanced CT scan showed lymph nodes swelling (yellow arrow). **(J)** PET-CT indicated abnormal accumulation (yellow arrow). **(K)** Hematoxylin and eosin staining of the resected tumor with magnification × 20.

A 47-year-old male visited our hospital because of recurrent dysphagia. Upper gastrointestinal endoscopy revealed multiple cauliflower-like lesions with pigmentation in the middle esophagus (**Figure 1A**). Immunohistochemistry staining of the tumor biopsy was positive for HMB-45, MelanA, SOX-10, and S-100, which confirmed diagnosis of PMME (**Figures 1B–F**). Contrast-enhanced CT scan (**Figures 1G–I**) showed wall thickening in the middle esophagus, and enlargement of right upper paratracheal and left gastric nodes according to the AJCC/UICC eighth edition staging manual of the esophagus and esophagogastric junction cancer (8). Positron emission tomography (PET)-CT indicated abnormal accumulation in the same region (**Figure 1J**), without obvious distant metastasis.

The patient underwent thoracoscopic and laparoscopic partial esophagectomy, two-field lymphadenectomy, as well as a left cervical esophagogastrostomy. The pathological examination (**Figure 1K**) demonstrated submucosal invasion of tumor cells (T1) with visible tumor thrombus in the blood vessels. A total of 63 lymph nodes were harvested. Among these, 4 nodes were positive for metastases (No. 2L: 0/2; No. 2R: 1/3; No. 4L: 0/7; No. 4R: 0/3; No. 7: 0/14; No. 8: 0/3; No. 15: 0/6; No. 16: 2/8; No. 17: 1/10; No. 18: 0/3; No. 19: 0/2; No. 20: 0/2 positive), indicating PMME (pT1N2M0, stage IIIB [AJCC 8^th^ (9)]). PD-L1 immunohistochemical staining was performed, revealing a Tumor Proportion Score (TPS) of 70%. The BRAF mutation analysis was negative, other driver mutations, including c-KIT and NRAS, were not tested due to the limited availability of targeted therapies for these mutations and the high cost of the testing platform in our clinical setting.

**Figure 2:**
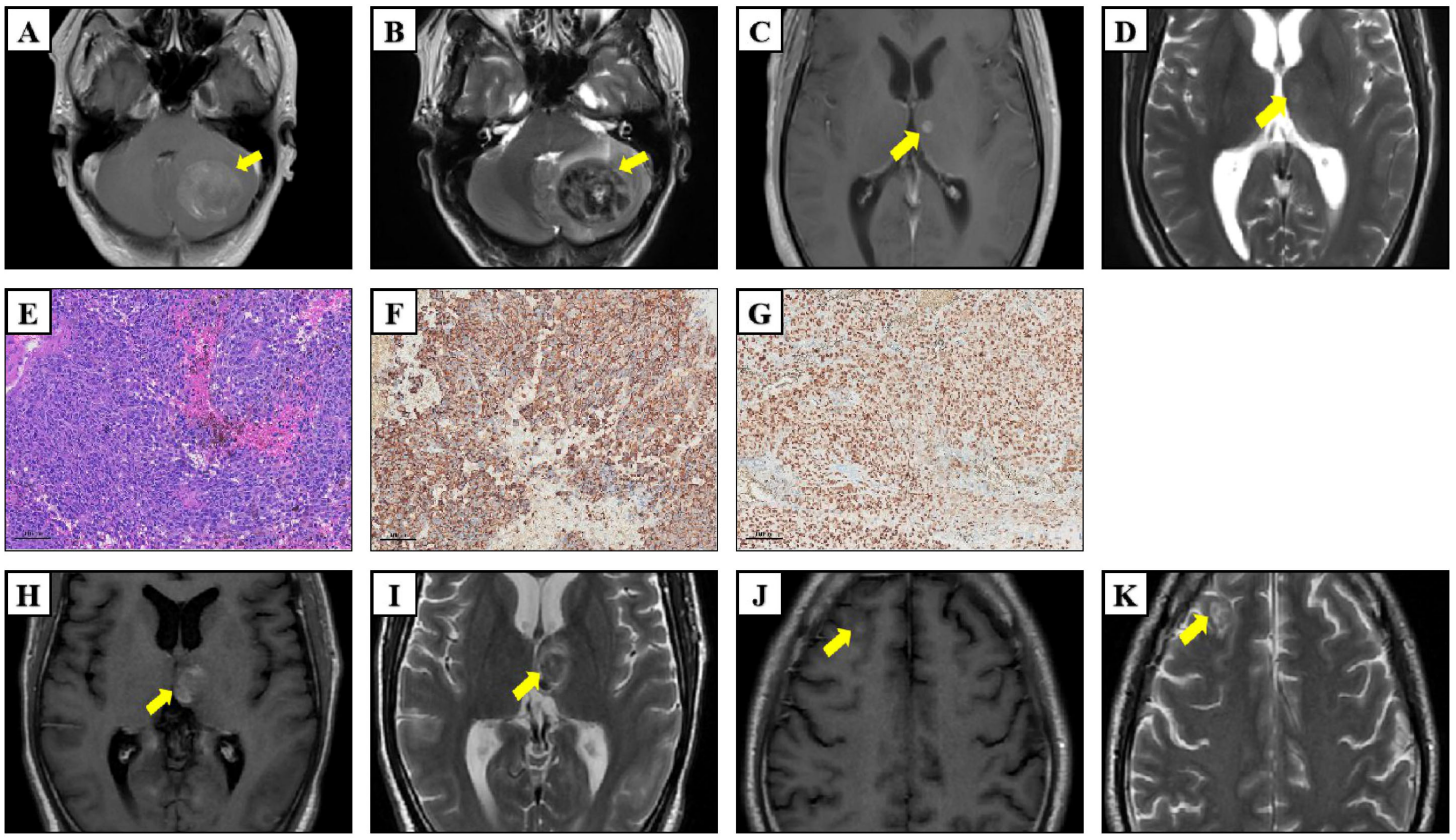
Brain MRI scan showed abnormal mass in the left cerebellum **(A, B)** and thalamus **(C, D)** (yellow arrow). Hematoxylin and eosin staining **(E)**, Immunohistochemistry staining of HMB-45 **(F)**, and Sox-10 **(G)** in the resected tumor with magnification × 40. Brain MRI scan showed an enlarged left thalamic nodule **(H, I)**, and a new nodule in the right frontal lobe **(J, K)** (yellow arrow).

**Figure 3:**
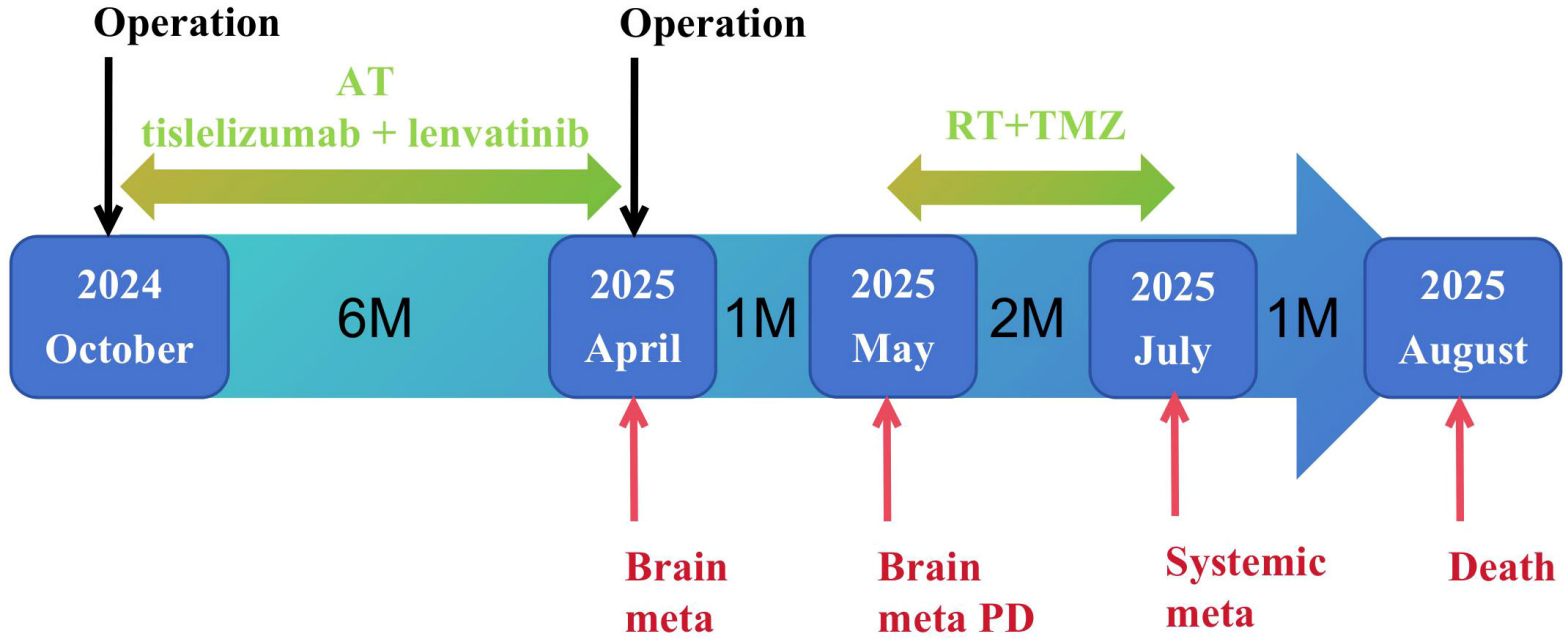
Timeline of the treatment interventions from surgery to metastasis and death. AT, adjuvant therapy; RT, radiation therapy; Meta, metastasis; PD, Progressive Disease; M, months; TMZ, temozolomide.

As adjuvant treatment, the patient refused cytotoxic chemotherapy and received five cycles of intravenous tislelizumab (200 mg, every 3 weeks) combined with oral lenvatinib (12 mg/day). Five months after the surgery, the patient experienced headaches, and a brain MRI scan showed abnormal mass in the left cerebellum and thalamus, indicating metastatic tumors (**Figures 2A–D**). The patient then underwent a tumor resection of the left cerebellum under navigational microscopy, and the pathological examination of the resected tissue identified metastatic malignant melanoma (**Figures 2E–G**). Three weeks after surgery, a brain MRI showed an enlarged left thalamic nodule and a new nodule in the right frontal lobe (**Figures 2H–K**). Stereotactic radiosurgery was initiated for these lesions at a dose of 35 Gy in 5 fractions, along with oral temozolomide. One month after radiotherapy, his condition deteriorated rapidly with systemic progression, and he eventually passed away ten months after diagnosis; the timeline of these events is shown in **Figure 3**.

## Discussion

PMME is a rare malignant tumor originating from epidermal melanocytes, accounting for only 0.1-0.3% of all esophageal malignancies (1, 3, 6, 10). Dysphagia is a common initial symptom, and as the disease progresses, patients may experience chest pain, weight loss, and upper gastrointestinal bleeding (2, 4, 6, 11). Upper gastrointestinal endoscopy is a primary diagnostic tool, typically showing submucosal protrusions with pigmentation in the middle and lower esophagus. Pathological examination combined with Immunohistochemistry is crucial for a definitive diagnosis. In this case, positive markers including HMB-45, Melan-A, S-100, and SOX-10 support the diagnosis of melanoma.

PMME is highly aggressive and metastatic, thus most patients are diagnosed at an advanced stage or even with metastasis (2, 4, 5). These patients are generally unresponsive to conventional treatments like chemotherapy and radiotherapy. Radical surgery remains the primary approach for PMME, as it maximizes tumor removal and reduces local recurrence risk (5–7). Therefore, this patient underwent radical surgery and extensive lymph node dissection shortly after diagnosis.

Recently, a large real-world study involving Asian patients with PMME demonstrated that lymph node metastasis is an independent poor prognostic factor for PMME (12). The 3-year relapse-free survival (RFS) rate for lymph node-positive patients was only 6.9%, compared with 57.8% for lymph node-negative patients. The postoperative pathology in our case confirmed the presence of lymph node metastasis, and the patient experienced extensive intracranial metastases as early as 5 months postoperatively, which was relatively uncommon in PMME (brain metastases accounted for only 3.8% in the Ishiguro cohort). This may suggest a uniquely aggressive biological behavior of this case and provides clues for further investigation into the metastatic pathways of PMME.

The biological behavior of PMME differs from cutaneous melanoma, making it more prone to vascular invasion and significantly increasing postoperative risks of recurrence and metastasis (5, 13), which further emphasizes the importance of adjuvant therapy. Current research indicates that for patients with BRAF mutations, the preferred adjuvant treatment is dabrafenib combined with trametinib (14). For patients without BRAF mutations, adjuvant chemotherapy demonstrates better efficacy than adjuvant interferon therapy (15, 16). In recent years, the application of immunotherapy has achieved significant breakthroughs in cancer treatment. Adjuvant PD-1 inhibitors could extend progression-free survival in mucosal melanoma patients, particularly for those with PD-L1 positive (17). Additionally, considering that mucosal melanoma is prone to vascular invasion, relevant studies have shown that the combination of VEGF inhibitors and PD-1 inhibitors demonstrates favorable outcomes in advanced mucosal melanoma (18, 19). Furthermore, Lili Mao et al. reported that the combination of pembrolizumab and lenvatinib has also achieved significant pathological response in neoadjuvant therapy (20). Ishiguro et al. also found that immunotherapy could achieve a median overall survival (OS) of 15.0 months and an objective response rate (ORR) of 23.5% in advanced PMME patients (12). In this case, the patient did not have a BRAF mutation and refused cytotoxic chemotherapy, so immunotherapy combined with anti-angiogenic therapy was initiated. However, this approach failed to effectively control tumor progression, leading to widespread brain metastasis 5 months after surgery, and subsequent systemic disease spread. As large-molecule therapeutic agents, PD-1 and VEGF inhibitors have poor blood-brain barrier (BBB) penetration, which created a specialized microenvironmental sanctuary that enables tumor cells to escape immune surveillance. This outcome suggests that for high-risk postoperative patients with node-positive disease, the current standard adjuvant therapy may be insufficient to effectively control micro metastases, particularly in preventing central nervous system relapse, and the benefit of such combination therapies may be highly selective.

The prognosis of PMME is poor, with a 5-year OS rate ranging from 2% to 17% and a median OS of 10–20 months (2, 5, 6). Patients with lower T stage and no lymph node metastasis generally have better prognosis (2, 6, 13), while those with stage III-IV disease show significantly poorer prognosis (21, 22). In this case, despite receiving radical surgery combined with adjuvant immunotherapy and anti-angiogenic therapy, the patient experienced rapid intracranial metastases, resulting in a final OS of only 10 months, consistent with the generally poor prognosis of PMME. However, the clinical value of this report lies in: first, demonstrating the insufficient efficacy of current PD-1-based adjuvant regimens in preventing brain relapse, that we attribute to the combination of BBB-limited drug penetration and the high-risk node-positive status; and second, underscoring the urgent need to explore novel combination treatment strategies and the resistance mechanisms underlying the aggressiveness of PMME and treatment failure. These findings provide clear direction for future research aimed at improving the prognosis of this severe disease.

## Conclusions

We report a rare case of a patient diagnosed with PMME, who experienced rapid intracranial metastases despite successful curative resection and adjuvant therapy, leading to his demise ten months after diagnosis. This highlights the exceptionally poor prognosis and highly metastatic potential of PMME, and the continued exploration of more effective therapeutic strategies remains essential to improve the prognosis and quality of life for future PMME patients.

## Data Availability

The original contributions presented in the study are included in the article/supplementary material. Further inquiries can be directed to the corresponding authors.
